# Contingency discrimination training and resurgence: Effects of reduced extinction session durations

**DOI:** 10.1002/jeab.70072

**Published:** 2025-12-29

**Authors:** Timothy A. Shahan, Joshua B. Hiltz, Matias Avellaneda, Brian D. Greer

**Affiliations:** ^1^ Utah State University Logan UT USA; ^2^ Children's Specialized Hospital–Rutgers University Center for Autism Research, Education, and Services (CSH–RUCARES) Somerset NJ USA; ^3^ Rutgers Brain Health Institute Piscataway NJ USA; ^4^ Rutgers Robert Wood Johnson Medical School New Brunswick NJ USA

**Keywords:** alternative reinforcement, extinction, lever press, matching law, rats, resurgence

## Abstract

Resurgence is an increase in a suppressed target behavior following a worsening of conditions for a more recently reinforced alternative behavior. Prior research shows that exposure to equal‐duration sessions of alternative reinforcement availability versus unavailability during treatment (i.e., contingency discrimination training; CDT) reduces resurgence. Clinically, minimizing exposure to extinction while maintaining the resurgence‐mitigating effects of CDT would be desirable. This experiment examined the effects of reduced off‐session durations by exposing groups of rats to different ratios of off:on session durations: All On (0 min: 30 min), CDT 1:1 (30 min: 30 min), CDT 1:2 (15 min: 30 min), CDT 1:6 (5 min: 30 min), and CDT escalate (i.e., [Esc] off‐session duration increased across sessions). Resurgence decreased exponentially with “off” session duration, with CDT 1:2 reducing resurgence and both CDT 1:1 and CDT Esc eliminating resurgence while generating control of alternative behavior by the prevailing reinforcement contingencies, without increasing the total number of target responses during treatment. Resurgence as choice in context theory described the data well with the assumption that the effect of the signaling properties of the reinforcement contingencies themselves increases linearly with the off:on duration ratio, as is true with the S−/S+ ratio in other discriminations.

Resurgence is an increase in a previously suppressed target behavior following a worsening of conditions (e.g., extinction) for a more recently reinforced alternative behavior (Lattal & Wacker, 2015; Shahan & Craig, 2017). Resurgence can be adaptive (Epstein, 1983; Shahan & Chase, 2002; Williams & St. Peter, 2020), but it can also generate relapse of undesirable behavior such as drug seeking (e.g., Podlesnik et al., 2006; Quick et al., 2011) or severe problem behavior following treatment with extinction plus differential reinforcement of alternative behavior (DRA; e.g., Briggs et al., 2018; Greer et al., 2024; Volkert et al., 2009; Wacker et al., 2011). Thus, it is important to identify techniques that may help to mitigate resurgence (Fisher et al., 2022; Greer & Shahan, 2019; Kimball et al., 2023).

Examinations of resurgence in clinical and laboratory settings generally use a three‐phase arrangement. In Phase 1 (i.e., baseline), reinforcers are delivered for some target behavior. In Phase 2 (i.e., treatment), target responses are placed on extinction, and an alternative response produces reinforcement (i.e., DRA). In Phase 3 (i.e., resurgence test), both the target and alternative responses are placed on extinction, and target behavior increases (i.e., resurgence).

Most laboratory attempts to identify manipulations during treatment that can effectively reduce resurgence have been met with limited success. For example, although lower rates or magnitudes of alternative reinforcement tend to reduce the size of target response‐rate increases during a subsequent resurgence test, the smaller increases are the result of less effective suppression of target behavior during treatment (e.g., Bouton & Trask, 2016; Craig & Shahan, 2016; Sweeney & Shahan, 2013). Another plausible approach, increasing the duration of treatment with extinction plus DRA, has similarly failed to generate robust decreases in resurgence (Nall et al., 2018; Winterbauer et al., 2013). Although a significant effect of treatment duration can be detected in the animal laboratory under highly controlled conditions (Shahan, Browning, & Nall, 2020), the reduction in resurgence is small enough to suggest no clinically relevant effect—an expectation confirmed in two clinical studies (Greer et al., 2020, 2023).

One treatment approach that has shown considerable promise in reducing resurgence in the laboratory is exposure to alternating sessions in which alternative reinforcement is available and then unavailable during target‐response extinction. Exposure to such cycling on/off alternative reinforcement reduces resurgence of both rats and humans as compared to exposure to consistently available alternative reinforcement (Schepers & Bouton, 2015; Shahan, Browning, & Nall, 2020; Shahan et al., 2024; Thrailkill et al., 2019; Trask et al., 2018; Wacker et al., 2011). Theoretical accounts of this effect have suggested that on/off alternative reinforcement teaches organisms to discriminate that the absence of alternative reinforcement signals the continued unavailability of reinforcement for target behavior, thus reducing subsequent resurgence (Schepers & Bouton, 2015; Shahan, Browning, & Nall, 2020). Given that such a discrimination is based not on arbitrary signaling stimuli but on the signaling properties of the reinforcement contingencies themselves, treatment with on/off alternative reinforcement has been referred to as *contingency discrimination training* (CDT; Shahan et al., 2024).

Although CDT has shown promise as a way to reduce resurgence, variations in the details of how the procedure is implemented remain relatively unexplored. Previous studies have systematically varied only the number of on/off cycles experienced during treatment (Schepers & Bouton, 2015; Shahan, Browning, & Nall, 2020). These studies have shown that as few as three cycles of on/off alternative reinforcement can substantially reduce subsequent resurgence, with increasing numbers of cycles showing relatively little added benefit.

In all previous examinations of CDT, the procedure has been implemented with alternating “on” and “off” sessions of equal duration. Thus, in the course of CDT, the alternative behavior is exposed to extinction for the same amount of time that it is reinforced. In the clinic, it is desirable to minimize exposure to extinction in general (Lerman et al., 1999) and especially to extinction of a desirable alternative response (e.g., appropriate communication). Thus, it is of considerable interest to determine if the resurgence‐mitigating effects of CDT might be maintained if shorter durations of exposure to extinction of the alternative behavior are arranged during treatment. Preserving the resurgence‐mitigating effects of CDT with shorter “off” session durations could improve the efficiency and practicality of implementations of CDT in the clinic.^1^

With explicit, arbitrary signaling stimuli, it is known that discrimination improves with longer durations of stimuli predictive of the absence of reinforcement (i.e., S−; Andrzejewski et al., 2007; Kalmbach et al., 2019). Further, Kalmbach et al. (2019) have shown that with a fixed S+ duration, discrimination performance increases linearly with the ratio of S−/S+ durations. Although CDT does not involve arbitrary signaling stimuli, the absence of alternative reinforcement itself during “off” sessions is thought to correspond to an S− period. As a result, shorter “off” sessions with a fixed “on” session duration might be expected to reduce the relevant discrimination and the efficacy of CDT. However, even if reductions in “off” session duration do reduce the efficacy of CDT, it is of considerable interest to determine whether the resurgence‐mitigating effects of CDT are preserved with relatively shorter “off” session durations.

Accordingly, the present experiment examined the effects of shorter “off” session durations during CDT on subsequent resurgence. Three groups of rats were exposed to a fixed “on” session duration with different “off” session durations across groups. Two additional groups allowed comparison with the effects of consistently available alternative reinforcement (i.e., all “on” sessions) and to “off” session durations that escalated throughout treatment (Phase 2).

## METHOD

### Subjects

Forty‐nine experimentally naïve male^2^ Long Evans rats (Charles River, Portage, MI) served as subjects. The original sample size was 50, but one subject was removed due to extremely low response rates during Phase 1. The rats were approximately 70–90 days old at the beginning of the experiment and were individually housed in a humidity‐ and temperature‐controlled colony room on a 12‐hr light : 12‐hr dark cycle (lights on at 0700 hours). Rats were provided with ad libitum access to water in their home cages and were maintained at 80% of their free‐feeding weights by supplemental feeding immediately after the session. All experimental procedures were conducted in accordance with the university's Institutional Animal Care and Use Committee.

### Apparatus

Ten identical Med Associates (St. Alban, VT) operant chambers (30 × 24 × 21 cm) were used. They were housed in sound‐ and light‐attenuating cubicles and included work panels on the right‐ and left‐side walls, with a clear Plexiglas ceiling, front door, and back wall. On top of the left‐side wall, a centered houselight provided general illumination. On the right‐side wall, two retractable levers with stimulus lights above them were positioned on either side of a receptacle that dispensed food (45‐mg grain‐based food pellets; Bio Serv, Flemington, NJ). Inside the room in which experimental sessions occurred, a computer running Med‐PC IV software controlled all experimental events and data collection.

### Procedure

Experimental sessions were conducted 7 days per week at approximately the same time each day and were conducted with five squads of 10 rats (counterbalanced across experimental groups) run sequentially, with start times separated by approximately 45 min. The first squad started at 0930 hours. Session duration was 30 min, except during Phase 2, when some groups experienced shorter “off” session durations as described below. Session durations excluded reinforcement deliveries, which were accompanied by a 3‐s receptacle illumination during which time all other stimuli were extinguished and all experimental timers paused. The reinforcer consisted of a single pellet delivery for all subjects.

#### Training

Four sessions of pellet‐magazine training were conducted prior to Phase 1. During these sessions, all chamber lights were off and both levers were retracted. Reinforcers were delivered on a variable‐time (VT) 60‐s schedule using 10 intervals derived from the Fleshler and Hoffman (1962) distribution (as were all VI schedules below).

#### Phase 1: Baseline

This phase began the day following the last magazine‐training session. One subject (LE 21) required supplemental lever‐press training after three sessions without acquiring the response. All other subjects did not require explicit lever‐press training. Sessions began with insertion of the target lever (left–right counterbalanced across rats) and illumination of the houselight and the stimulus light above the target lever. The first lever press on the target lever during the first session produced a pellet delivery. For the remainder of the first session and subsequent Phase 1 sessions, responses were reinforced on a VI 10‐s schedule. This phase lasted for 25 sessions for all subjects.

#### Phase 2: Treatment

At the end of Phase 1, subjects were assigned to one of five groups: All On (*n* = 10), CDT 1:1 (*n* = 10), CDT 1:2 (*n* = 10), CDT 1:6 (*n* = 10), and CDT Esc (*n* = 9) such that target response rates during the last 3 days of Phase 1 were similar between groups. All groups (except for All On, which experienced only “on” sessions) alternated between “on” and “off” sessions, with the first session being “on” for all groups. During “on” sessions, the target lever was placed on extinction and the alternative lever was reinforced on a VI 10‐s schedule. For all groups, “on” sessions always lasted 30 min. For “off” sessions, both the target and alternative lever were placed on extinction and the session duration was dependent on group assignment—where the ratios in group names refer to the ratio of off‐session to on‐session durations (e.g., 1:6 refers to 5‐min “off” sessions and 30‐min “on” sessions). Session durations for all groups during Phase 2 are shown in Table 1. Phase 2 sessions began with insertion of both levers and illumination of the houselight and both lever stimulus lights. A 3‐s changeover delay was arranged between target and alternative responding such that the alternative response would not produce an arranged reinforcer for 3 s following a target response. Phase 2 lasted 13 sessions for all subjects, with the 13th session corresponding to an “on” session for all groups.

**TABLE 1:** Session duration (in minutes) for each group across Phase 2.

Group	Session												
	1	2	3	4	5	6	7	8	9	10	11	12	13
All On	30	30	30	30	30	30	30	30	30	30	30	30	30
CDT 1:1	30	**30**	30	**30**	30	**30**	30	**30**	30	**30**	30	**30**	30
CDT 1:2	30	**15**	30	**15**	30	**15**	30	**15**	30	**15**	30	**15**	30
CDT 1:6	30	**5**	30	**5**	30	**5**	30	**5**	30	**5**	30	**5**	30
CDT Esc	30	**5**	30	**10**	30	**15**	30	**20**	30	**25**	30	**30**	30

*Note*: Sessions alternated between “on” and “off” except for the “All An” group that experienced only “on” sessions. The first session for all groups was “on.” “Off” session durations are in bold.

#### Phase 3: Resurgence test

Alternative responding for all subjects was placed on extinction for all sessions of Phase 3 while target responding remained on extinction. All other stimulus conditions remained as in Phase 2. Phase 3 consisted of seven 30‐min sessions for all subjects.

### Data analysis

Statistical analyses were conducted in R v4.4.0 (rstatix package), were two‐tailed with α = .05, and employed Greenhouse–Geiser corrections whenever sphericity violations were detected.

## RESULTS

Target responses/minute averaged across the last three sessions of Phase 1 for the All On, CDT 1:1, CDT 1:2, CDT 1:6, and CDT Esc groups were 47.24, 47.22, 46.85, 47.61, and 47.23, respectively, and did not differ significantly across groups, *F*(4, 44) = 0.002, *p* = 1.0, $\eta_{G}^{2}$ = .000.

**FIGURE 1:**
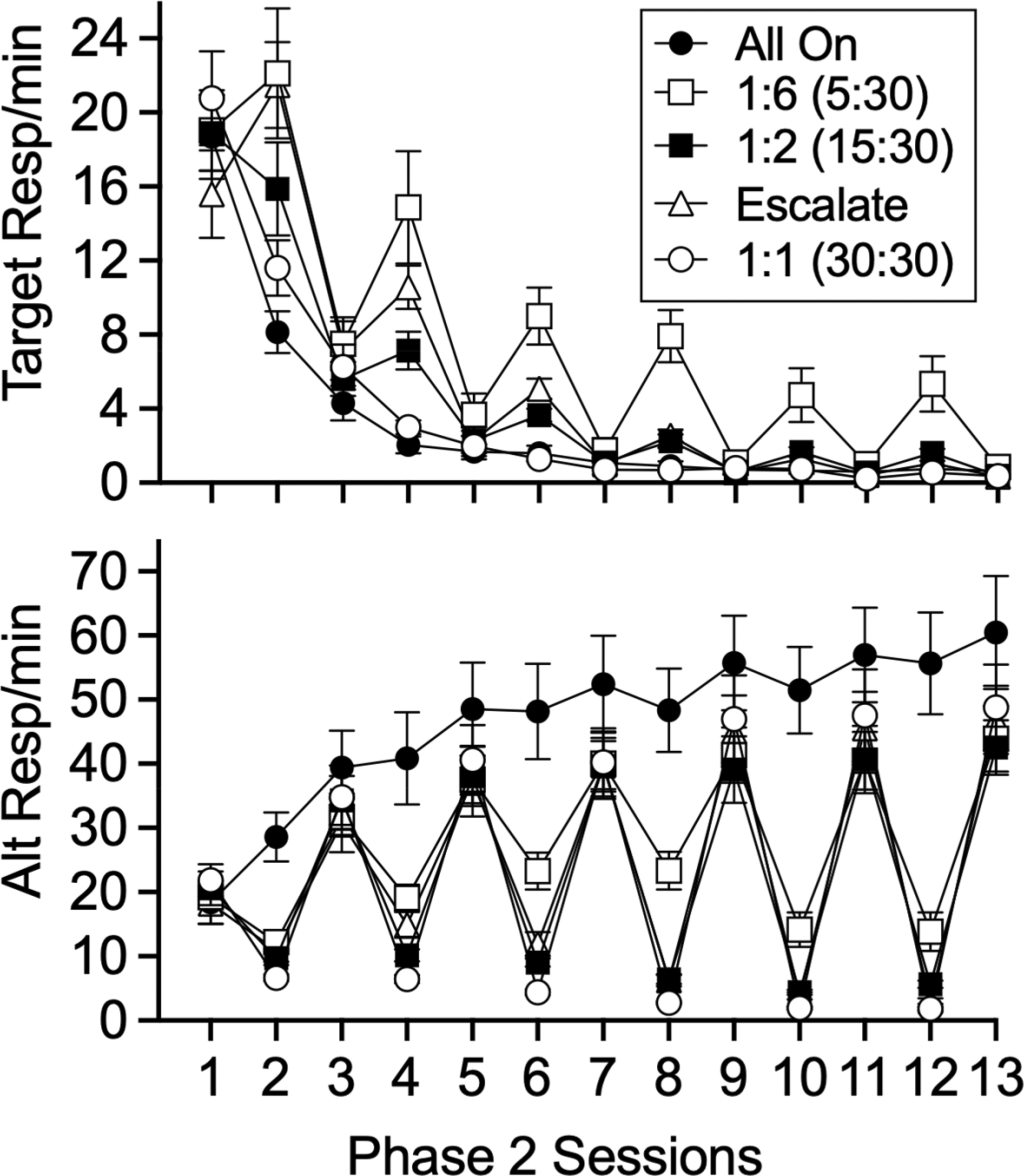
Mean target and alternative response rates during Phase 2. The top panel shows target response rates in all sessions of Phase 2 for each group. The bottom panel shows alternative response rates across all sessions of Phase 2. Error bars reflect ± SEM.

The top panel of Figure 1 shows mean target response/minute for all groups across sessions of Phase 2. Generally speaking, response rates decreased across sessions of Phase 2 and tended to be higher and decrease less for groups exposed to CDT. A Group × Session mixed ANOVA revealed that target response rates decreased significantly across sessions, *F*(2.63, 115.86) = 178.110, *p* < .001, $\eta_{G}^{2}$= .727, were different for the groups, *F*(4, 44) = 6.405, *p* < .001, $\eta_{G}^{2}$ = .166, and decreased differentially across sessions for the different groups, *F*(10.53, 115.86) = 5.058, *p* < .001, $\eta_{G}^{2}$ = .232. A separate Group × Session × “On” versus “Off” mixed ANOVA restricted to groups experiencing “off” sessions (i.e., all groups exposed to CDT) revealed a significant three‐way interaction, *F*(6.75, 78.70) = 5.557, *p* < .001, $\eta_{G}^{2}$ = .058. The interaction resulted from the fact that response rates tended to be higher for “off” sessions (i.e., even numbered sessions) than for “on” sessions (i.e., significant main effect of “on” vs. “off”), *F*(1, 35) = 12.074, *p* = .001, $\eta_{G}^{2}$ = .035, more so for groups with shorter “off” sessions (i.e., significant Group × “On” vs. “Off” interaction), *F*(3, 35) = 13.048, *p* < .001, $\eta_{G}^{2}$ = .106, and tended to decrease less across sessions for “off” than for “on” sessions (i.e., significant Session × “On” vs. “Off” interaction), *F*(2.25, 78.90) = 5.311, *p* = .005, $\eta_{G}^{2}$ = .019.

The bottom panel of Figure 1 similarly shows mean alternative responses/minute across all sessions of Phase 2. Response rates increased across sessions of Phase 2 and tended to be lower and increase less for groups exposed to CDT. A Group × Session mixed ANOVA revealed that alternative response rates increased significantly across sessions, *F*(2.22, 97.85) = 148.107, *p* < .001, $\eta_{G}^{2}$ = .495, were different for the groups, *F*(4, 44) = 6.895, *p* < .001, $\eta_{G}^{2}$ = .308, and increased differentially across sessions for the different groups, *F*(8.90, 97.85) = 10.181, *p* < .001, $\eta_{G}^{2}$ = .212. A separate Group × Session × “On” versus “Off” mixed ANOVA restricted to groups experiencing “off” sessions revealed a significant three‐way interaction, *F*(6.61, 77.11) = 3.118, *p* = .007, $\eta_{G}^{2}$ = .018, resulting from the fact that response rates tended to be lower for “off” sessions (i.e., even numbered sessions) than for “on” sessions (i.e., significant main effect of “on” vs. “off”), *F*(1, 35) = 193.351, *p* < .001, $\eta_{G}^{2}$ = .626, less so for groups with shorter “off” sessions (i.e., significant Group × “On” vs. “Off” interaction), *F*(3, 35) = 3.493, *p* < .026, $\eta_{G}^{2}$ = .083, and tended to increase less across sessions for “off” than for “on” sessions (i.e., significant Session × “On” vs. “Off” interaction), *F*(2.20, 77.11) = 106.261, *p* < .001, $\eta_{G}^{2}$ = .018.

**FIGURE 2:**
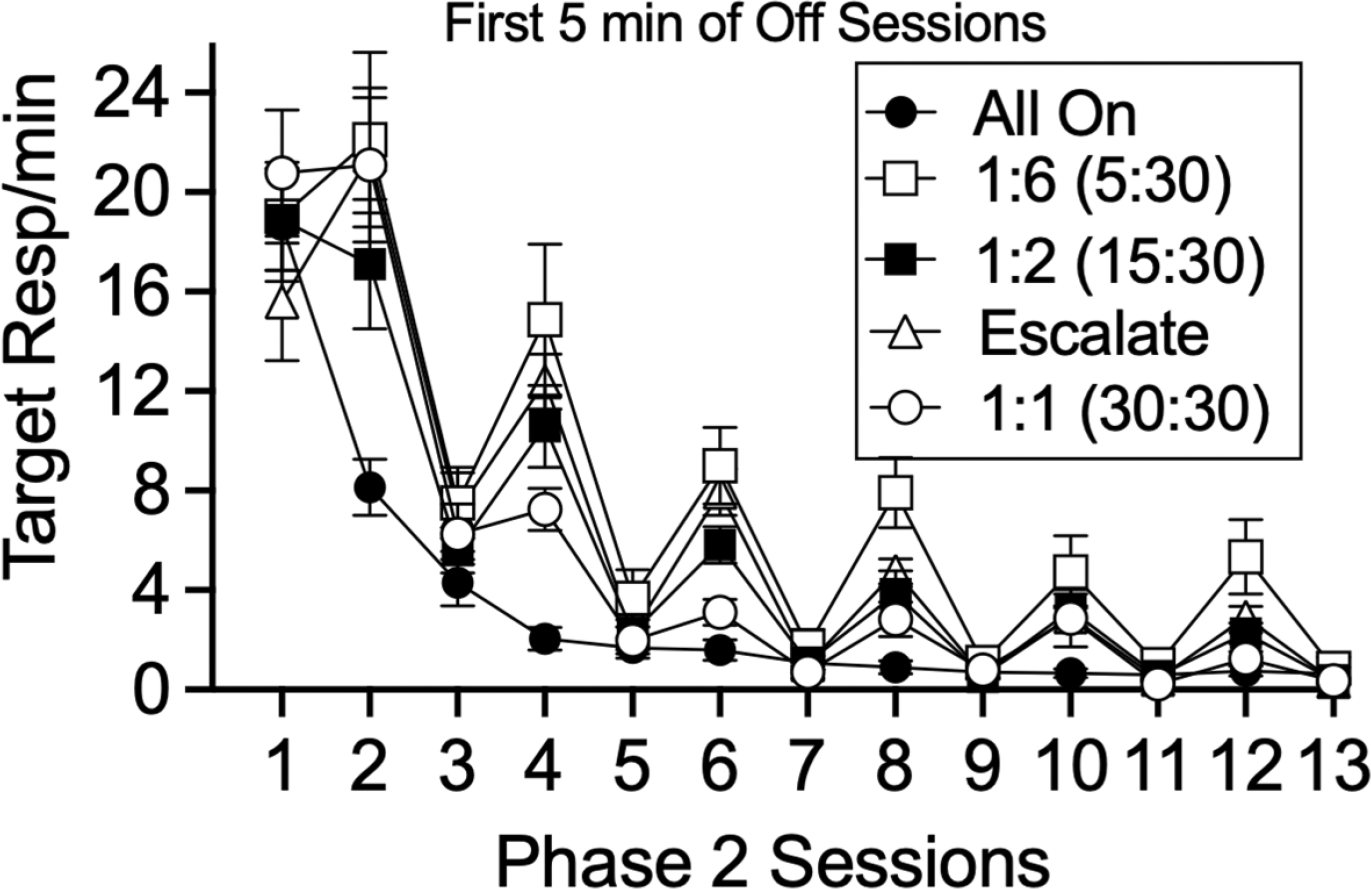
Mean target response rates during Phase 2 across the entire session for “on” sessions and the first five minutes for “off” sessions. Error bars reflect ± SEM.

One notable feature of target response rates in the top panel of Figure 1 is that they tended to be higher during “off” sessions when those sessions were shorter. However, it is important to note that the longer “off” sessions provided a larger sample of target responding (i.e., more time for target responding to occur). Because of this difference, lower response rates during the extinction sessions for the longer “off” session groups could have resulted from the inclusion of extra session time during which little target responding occurred. Thus, it is of some interest to see if response rates differed for the different CDT groups when compared within a similar sample of time. All CDT groups experienced “off” sessions of at least 5 min, and thus Figure 2 shows the same data as the top panel of Figure 1, except that target response rates were calculated using only the first 5 min of the “off” sessions for all CDT groups. Even when examined using a similar first‐5‐min time base, response rates tended to be higher for CDT groups exposed to shorter “off” session durations. A Group × Session × “On” versus “Off” mixed ANOVA restricted to groups experiencing “off” sessions (i.e., all groups exposed to CDT) revealed significant main effects of session, *F*(1.46, 51.14) = 194.349, *p* < .001, $\eta_{G}^{2}$ = .690; “on” versus “off,” *F*(1, 35) = 52.062, *p* < .001, $\eta_{G}^{2}$ = .128; a significant Group × “On” versus “Off” interaction, *F*(3, 35) = 3.768, *p* = .019, $\eta_{G}^{2}$ = .031; and a significant Session × “On” versus “Off” interaction, *F*(2.52, 88.33) = 3.733, *p* = .019, $\eta_{G}^{2}$ = .013. Thus, the higher target response rates during shorter “on” sessions were not entirely due to the use of a differential time base for those sessions in Figure 1.

**FIGURE 3:**
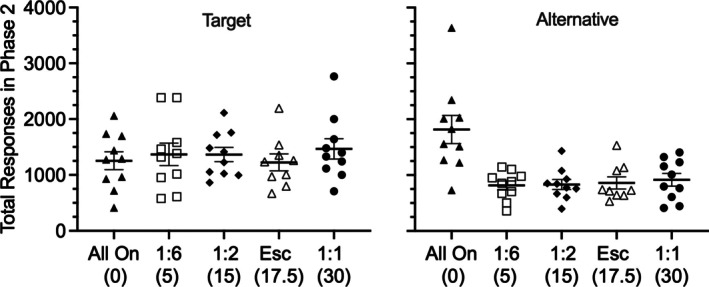
Mean total number of target and alternative responses during Phase 2. The left panel shows the total number of target responses emitted in all sessions of Phase 2. The right panel shows the total number of alternative responses emitted across all sessions of Phase 2. Numbers in parentheses below the *x*‐axis labels reflect the “off” session duration, with zero representing the absence of “off” sessions for the “All On” group. Each point represents the data from one individual rat. Error bars reflect ± SEM.

Perhaps more important clinically than the *rate* of target responding across sessions in Phase 2 is the *total number* of occurrences of the target behavior across the entirety of the different treatments (e.g., Smith & Greer, 2023). For example, if the target behavior was severe problem behavior, then it would be important to know if the treatments differed in their overall number of occurrences throughout treatment. To examine this issue, the left panel of Figure 3 shows that the total (i.e., cumulative) number of target responses recorded across all sessions of Phase 2 (including both “on” and “off” sessions where relevant) did not differ across groups (i.e., there was no significant effect of group), *F*(4, 44) = 0.34, *p* = .85, $\eta_{G}^{2}$ = .03. A similar question might be asked about the alternative behavior given that in the clinic such behavior would be desirable. Thus, the right panel of Figure 3 shows a similar analysis of the total number of alternative responses recorded across all sessions of Phase 2. In this case, there was a significant effect of group, *F*(4, 44) = 9.056, *p* < .001, $\eta_{G}^{2}$ = .452, driven entirely by the greater number of alternative responses for the All On group, a result that is not surprising given that this was the only group for which alternative behavior was reinforced across all sessions of Phase 2.

**FIGURE 4:**
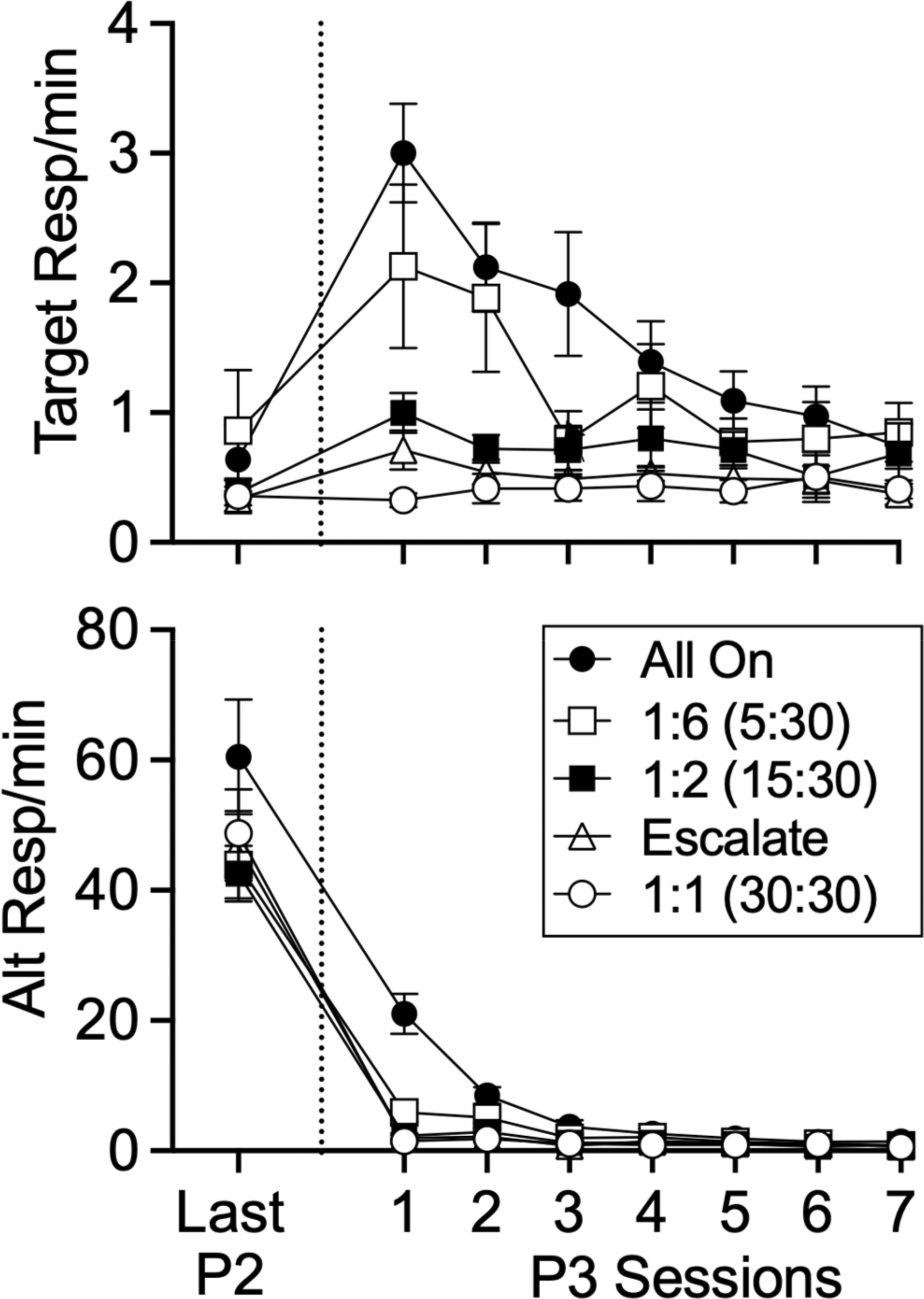
Mean target and alternative response rates in the last session of Phase 2 and in all sessions of Phase 3 (resurgence test). Error bars reflect ± SEM.

**FIGURE A1:**
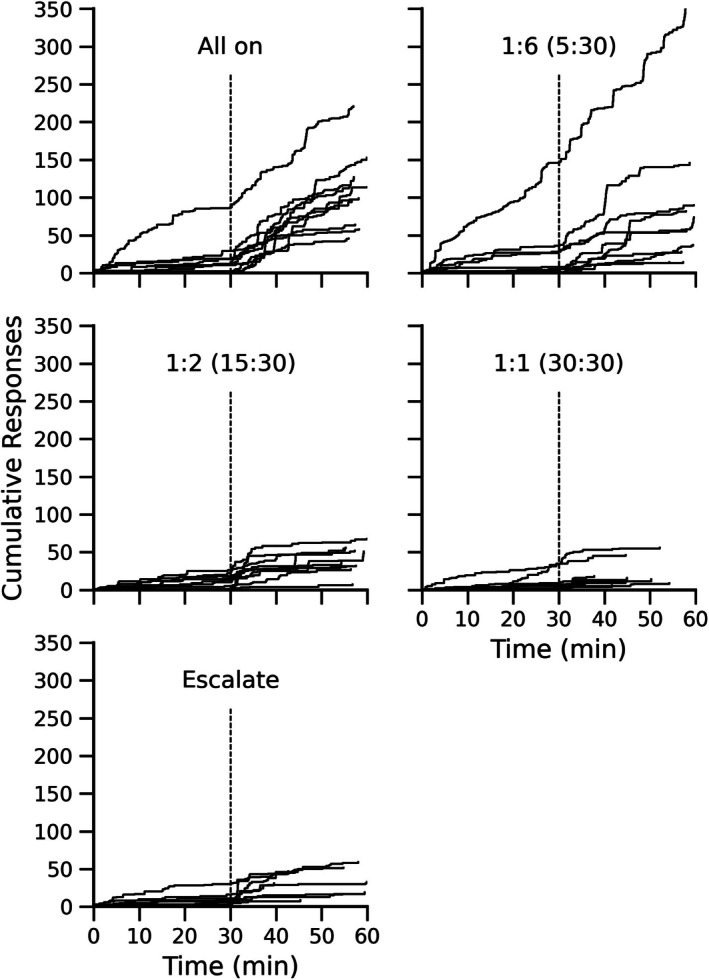
Cumulative target responses across the last session of Phase 2 and first session of Phase 3. Individual rats are represented by different lines. The vertical dashed line represents the transition between Phase 2 and Phase 3. Data are presented separately for each group.

The top panel of Figure 4 shows mean target responses/minute for all groups in the last session of Phase 2 and in all sessions of the Phase 3 resurgence test. Target rates for all groups were low in the final session of Phase 2 (an “on” session for all groups) and increased differentially across groups in the first session of Phase 3. A Group × Phase mixed ANOVA comparing the last session of Phase 2 with the first session of Phase 3 revealed significant main effects of group, *F*(4, 44) = 5.24, *p* = .002, $\eta_{G}^{2}$ = .286, and phase *F*(4, 44) = 69.65, *p* < .001, $\eta_{G}^{2}$ = .201, and a significant Group × Phase interaction, *F*(4, 44) = 14.65, *p* < .001, $\eta_{G}^{2}$ = .174. Tukey's HSD was used to construct all pairwise comparisons for groups within and between Phases 2 and 3, controlling for the familywise error rate. Although none of the groups differed from any of the others in the last session of Phase 2—all *t*s(44) < 1.405, *p*s > .628—in the first session of Phase 3, the All On group showed significantly higher target rates than all CDT groups—*t*s(44) > 4.087, *p*s < .0016—except for the CDT 1:6 group, *t*(44) = 1.786, *p* = .3946. Thus, all CDT groups except for the CDT 1:6 group with the shortest (i.e., 5 min) “off” session showed reduced resurgence relative to the All On group for which alternative reinforcement was always available in Phase 2. Further, focusing on the different CDT groups, the CDT 1:6 group differed significantly from the CDT 1:1 group, *t*(44) = 3.666, *p* < .006, and marginally from the CDT Esc group, *t*(44) = 2.806, *p* = .0547, but no other group differences were significant, *t*s(44) < 2.302, *p*s > .164. Finally, focusing on the change in response rates from the last session of Phase 2 to the first session of Phase 3 for all groups, the All On, *t*(44) = 9.719, *p* < .001; CDT 1:6, *t*(44) = 5.202, *p* < .001; and the CDT 1:2, *t*(44) = 2.519, *p* = .0155, groups showed significant increases in responding. Neither the CDT 1:1, *t*(44) = 0.123, *p* = .903, nor the CDT Esc, *t*(44) = 1.472, *p* = .148, groups showed a significant increase in responding. In summary, except for the shortest (i.e., 5 min) “off” session duration, shorter “off” sessions as part of CDT reduced resurgence relative to all on alternative reinforcement, with both the longest “off” duration (i.e., 30 min for the CDT 1:1 group) and escalating “off” durations eliminating resurgence. The intermediate, 15‐min “off” session duration arranged for the CDT 1:2 group reduced resurgence significantly but did not eliminate it. Appendix Figure A1 shows cumulative records for individual rats across groups in the last session of Phase 2 and the first session of Phase 3. The cumulative records suggest that the increases in responding for the “All On” and CDT 1:6 groups were consistent across much of the first session of Phase 3, but increases in responding for the other groups, when they occurred (i.e., for the CDT 1:1 and CDT Esc groups), tended to be more restricted to the first 10 min of the session.

Next, considering target response rates across all seven Phase 3 sessions in the top panel of Figure 4, target responding tended to be higher for the All On and CDT 1:6 groups than for the other groups and it tended to decrease more across sessions. Consistent with this summary, a Group × Session mixed ANOVA showed main effects of group, *F*(4, 44) = 7.272, *p* < .001, $\eta_{G}^{2}$ = .264, and session, *F*(3.21, 141.11) = 12.887, *p* < .001, $\eta_{G}^{2}$ = .118, and a significant Group × Session interaction, *F*(12.83, 141.11) = 4.087, *p* < .001, $\eta_{G}^{2}$ = .145.

The bottom panel of Figure 4 shows mean alternative responses/minute for all groups in the last session of Phase 2 and in all sessions of the Phase 3 resurgence test. Alternative responding was higher and decreased more with the Phase 2 to 3 transition for the All On group than for the other groups. A Group × Phase mixed ANOVA comparing the last session of Phase 2 with the first session of Phase 3 revealed a significant main effect of group, *F*(4, 44) = 25.31, *p* < .001, $\eta_{G}^{2}$ = .637, and phase, *F*(1, 44) = 19.84, *p* < .001, $\eta_{G}^{2}$ = .097, and a significant Group × Phase interaction, *F*(4, 44) = 7.38, *p* < .001, $\eta_{G}^{2}$ = .320. Response rates were similarly higher for the “All On” group and decreased more when considering all seven sessions of Phase 3. Considering alternative response rates across all Phase 3 sessions, a Group × Session mixed ANOVA revealed a significant main effect of group, *F*(4, 44) = 16.507, *p* < .001, $\eta_{G}^{2}$ = .379, session *F*(1.61, 71.03) = 55.901, *p* < .001, $\eta_{G}^{2}$ = .429, and a significant Group × Session interaction, *F*(6.46, 71.03) = 22.348, *p* < .001, $\eta_{G}^{2}$ = .546.

**FIGURE A2:**
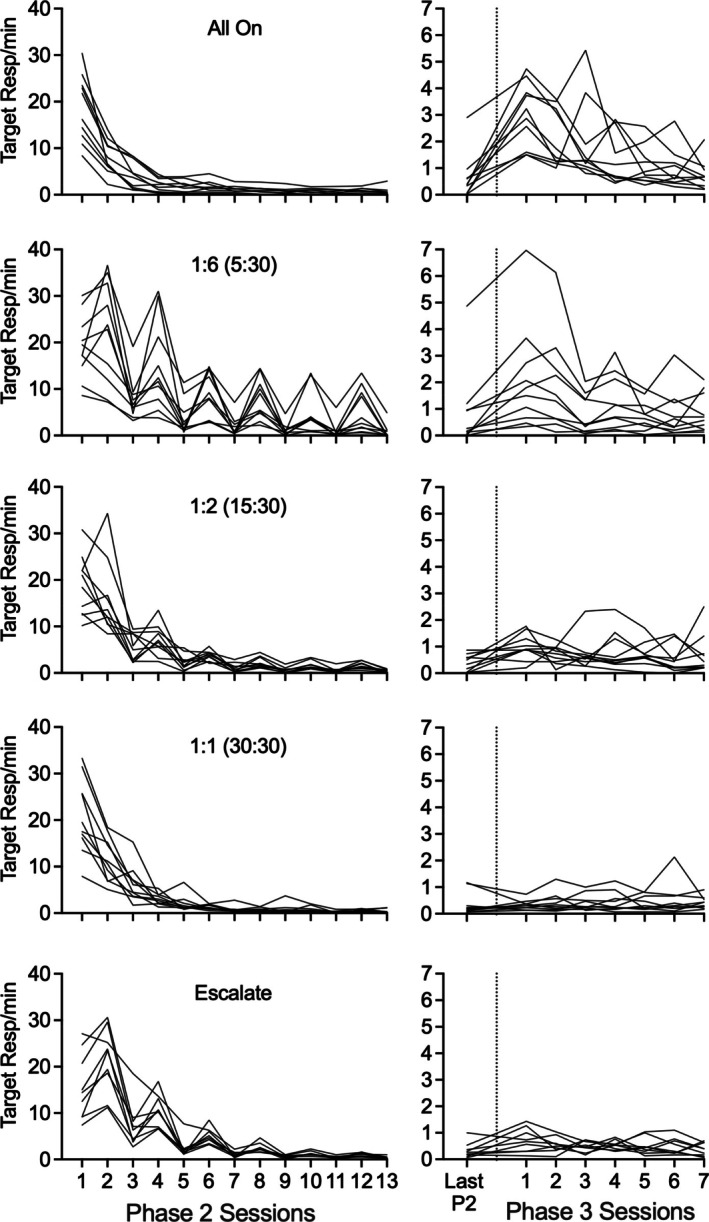
Target response rates for individual rats. The left column shows target responding across all sessions of Phase 2. The right column shows responding in the last session of Phase 2 (i.e., Last P2) and across all sessions of the resurgence test in Phase 3 (i.e., Phase 3 sessions). Data are presented separately for each group.

Appendix Figure A2 shows that the patterns in mean target behavior for Phases 2 and 3 described above in Figures 1 and 4 are representative of the behavior of the individual rats within the groups.

## DISCUSSION

As in previous studies, the present experiment showed that exposure to a treatment (i.e., CDT) involving alternating sessions with alternative reinforcement available (i.e., “on”) versus unavailable (i.e., “off”) during extinction of a target behavior reduced subsequent resurgence relative to a condition in which alternative reinforcement was available throughout extinction (e.g., Schepers & Bouton, 2015; Shahan, Browning, & Nall, 2020; Shahan et al., 2024; Smith & Greer, 2023; Thrailkill et al., 2019; Trask et al., 2018). Importantly, the present experiment also showed that the resurgence‐mitigating effect of CDT can be fully or partially maintained under some conditions when exposure to extinction of the alternative behavior is reduced by shortening the duration of the “off” sessions.

One somewhat surprising finding in the present experiment was that although target response rates across Phase 2 tended to be higher for “off” versus “on” sessions for most CDT groups, this was not the case for the group experiencing equal‐duration 30‐min “off” and “on” sessions (i.e., the CDT 1:1 group). All previous examinations of CDT with rats have used equal‐duration 30‐min “on” and “off” sessions and have observed higher target response rates in “off” sessions. One notable difference between the present experiment and previous experiments with rats is that all previous experiments arranged a VI 30‐s schedule for target behavior during Phase 1 and a VI 10‐s schedule for the alternative behavior during “on” sessions in Phase 2. In the present experiment, a VI 10‐s schedule was arranged for the target behavior during Phase 1 and for the alternative behavior during Phase 2 “on” sessions. We chose to arrange a higher rate of reinforcement for target behavior in Phase 1 than in previous studies with rats to expand the range of Phase 1 reinforcement rates examined in studies of CDT and to better approximate the denser reinforcement schedules typically arranged in clinical settings (e.g., fixed‐ratio 1 for target responding in baseline). Interestingly, the lack of an increase in target response rates during “off” sessions for the CDT 1:1 group is consistent with the data from the extinction control groups of Craig and Shahan (2016). In their study, target response rates were lower in Phase 2 following training in Phase 1 with a higher than with a lower reinforcement rate, but only for groups for which extinction was arranged for the alternative behavior in Phase 2. Thus, the lack of sessionwide increases in target‐response rate in the present experiment during “off” sessions for the CDT 1:1 group likely reflects the fact that a higher Phase 1 reinforcement rate was arranged prior to experiencing extinction associated with the “off” sessions in Phase 2. Notably, increases in target‐response rate were observed in the present experiment for CDT groups experiencing shorter “off” session durations and for the CDT 1:1 group when the calculation of response rates was restricted to the first 5 min of the “off” sessions. Thus, the duration of exposure to extinction during “off” sessions can affect whether target response rates show an increase in “off” sessions in part by changing the time base used in the calculation of those rates, likely because longer “off” sessions result in the inclusion of additional session time during which less target responding occurs. Nevertheless, restricting the calculation of target response rates to the first 5 min of “off” sessions across groups did not eliminate differences in “off” session target rates across groups. Thus, the experience of different extinction‐session lengths itself also seems to affect target rates during “off” sessions across Phase 2. As a result, the rate of target behavior obtained during the periodic extinction exposures arranged by CDT seems likely to result from interactions between the rate of reinforcement arranged for target behavior in Phase 1 and the duration of the arranged extinction exposures. It also seems reasonable that the rate of reinforcement during “on” sessions of CDT might also interact with Phase 1 reinforcement rate, Phase 2 “off” session duration, or both. Future research might usefully examine these potential interactions more fully.

The above considerations about target response rates during “off” sessions notwithstanding, a more clinically relevant consideration is likely the total number of occurrences of the target behavior across the entirety of treatment (i.e., Phase 2). Smith and Greer (2023) showed with crowdsourced human participants that although CDT produced the characteristic cycling between low and high target response rates during “on” and “off” sessions, respectively, the total number of target responses during Phase 2 was lower for CDT than for conditions arranging consistently available (i.e., “all on”) alternative reinforcement. In the present experiment, we obtained no difference in total target responses across Phase 2 between groups. Although total number of target responses was not lower for the CDT groups, importantly, it also was not higher. Thus, resurgence was eliminated for the CDT 1:1 and CDT Esc groups and substantially reduced for the CDT 1:2 group despite that no additional target behavior was generated across treatments. If such findings were replicated in the clinic, it would suggest that there is little additional risk associated with the periodic exposures to extinction arranged by CDT—despite the increased benefit of the intervention in terms of mitigating resurgence. A similar analysis of alternative behavior during Phase 2 showed that there were more total occurrences of that behavior during treatment with “all on” alternative reinforcement than for all of the CDT conditions. Given that the alternative behavior was only reinforced in half of the Phase 2 sessions for the CDT groups, this result is not surprising. One potential concern with CDT could be this reduced number of occurrences of the alternative (i.e., desirable) behavior. However, the data suggest that this concern is misplaced, as the alternative behavior readily came under discriminative control of the contingencies in place, quickly reducing when reinforcement was unavailable for that behavior and then rebounding to high rates when reinforcement was again available. Such an outcome could be highly desirable in a naturalistic situation in which reinforcement for appropriate, alternative behavior is likely to be only occasionally unavailable. Thus, for alternative behavior, the total number of occurrences across treatment is likely to be less important than the degree to which that behavior shows *appropriate* and direct control by the contingencies in effect for the alternative behavior at a particular time.

**FIGURE 5:**
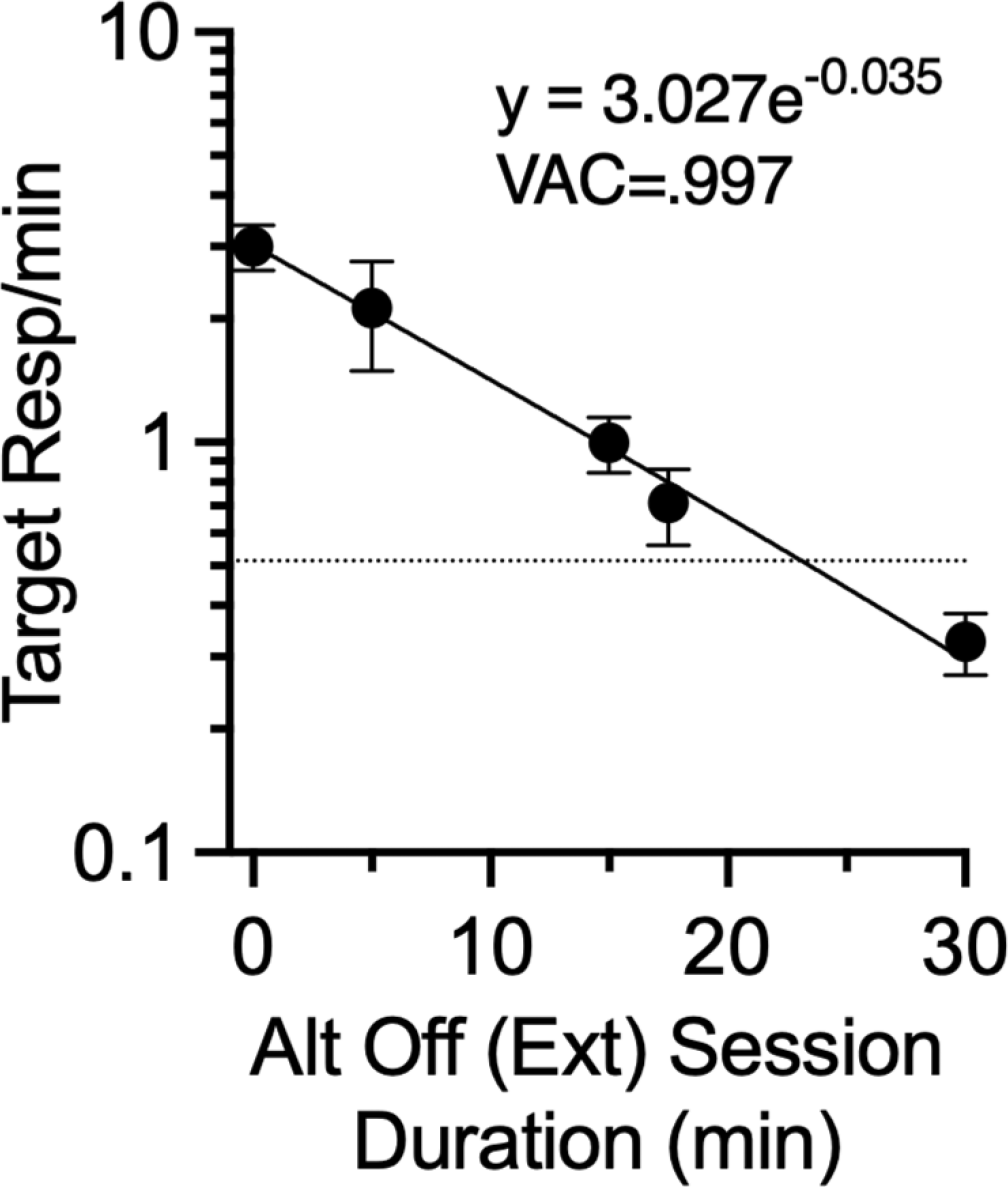
Mean target response rate during the first session of Phase 3 as a function of the average duration of “off” sessions. Each point represents the average data from one group. The expression of the function describing the data and the percentage of variance accounted for (VAC) are included in the panel (notice the logarithmic *y*‐axis). The dashed line represents the mean target response rate across groups in the last Phase 2 session. Error bars reflect ± SEM.

The discussion above suggests that CDT appears to have desirable characteristics during treatment in terms of avoiding production of more undesirable target behavior while also generating direct control of a desirable alternative behavior by the contingencies in effect. In addition to having these desirable characteristics during treatment, CDT mitigates subsequent resurgence. The primary focus of the present study was whether such mitigation might be maintained while reducing overall exposure to extinction of the alternative behavior during treatment by reducing “off” session durations. Figure 5 shows a summary of the effects of shortened “off” session durations by plotting the relation between resurgence (i.e., target response rates in the first session of Phase 3) and the average “off” session duration arranged during treatment.^3^ The dashed horizontal line reflects target response rates averaged across groups (which did not differ statistically) in the final session of Phase 2. The data points above 0, 5, 15, 17.5, and 30 on the *x*‐axis correspond to the All On, CDT 1:6, CDT 1:2, CDT Esc, and CDT 1:1 groups, respectively. The decrease in target response rates is well described by an exponential function (note the logarithmic *y*‐axis). Clinically, perhaps more important than the decrease in resurgence with increases in “off” session duration is the fact that due to the exponential nature of the function, even rather large decreases in “off” session duration produce modest absolute increases in target behavior. Even a 50% decrease in total exposure to extinction for the CDT 1:2 group produced only a modest increase in target behavior, and the slight increase in exposure to extinction associated with the CDT Esc condition produced elimination of resurgence statistically comparable to that of the CDT 1:1 condition. This finding suggests that the CDT Esc condition could be especially promising clinically where resurgence mitigation is highly desirable but practical considerations may require initially short extinction exposures when conducting CDT. However, it is important to note that it is impossible to tell at present if the escalation in “off” session duration plays any meaningful role in the success of the procedure. The reason is that CDT Esc was associated with a higher mean “off” session duration than the CDT 1:2 condition and produced a decrease in resurgence that is roughly consistent with what would be expected with that increased exposure to extinction based on the exponential function. If escalation of “off” session duration per se had an added effect, it appears that effect is rather modest. Future research could examine this issue by comparing various CDT escalation conditions to fixed “off” session conditions arranging the same average session duration or total exposure to extinction.

In addition to the clinical concerns noted above, the rationale for the present experiment was based in part on theoretical considerations about how shortened “off” session durations may affect the efficacy of CDT by altering the signaling properties of the reinforcement contingencies themselves. Specifically, resurgence as choice in context theory (i.e., RaC2; Shahan, Browning, & Nall, 2020) incorporates the suggestion from context theory (e.g., Schepers & Bouton, 2015) that exposure to on/off alternative reinforcement during Phase 2 teaches organisms two discriminations: (1) During “on” sessions, the availability of alternative reinforcement signals that reinforcement for target behavior is unavailable, and (2) during “off” sessions, the absence of reinforcement for the alternative signals unavailability of reinforcement for both options. As a result, the organism learns that the unavailability of reinforcement for the alternative response does not mean that reinforcement is available for the target response, thus serving to inoculate against resurgence later.

Note that within CDT, “on” sessions as signaled by the availability of alternative reinforcement might be considered to correspond to S+ periods, and “off” sessions as signaled by the absence of any reinforcer deliveries might be considered to correspond to S− periods. Based on the fact that in discriminations of arbitrary stimuli, longer S− periods produce increases in discrimination (Andrzejewski et al., 2007; Kalmbach et al., 2019), we reasoned that shorter “off” session durations might reduce discrimination of the relevant contingencies and thus potentially reduce the efficacy of CDT. The findings of the present experiment were generally consistent with this expectation. In what follows, we show that RaC2 provides a good quantitative description of the results of the present experiment.

RaC2 (see Shahan, Browning, & Nall, 2020, for a more detailed exposition) suggests that resurgence results from the same basic processes governing choice in general. It is based on the concatenated matching law (Baum & Rachlin, 1969; Herrnstein, 1961, 1970) and suggests that the rates of target and alternative behaviors are governed by the changing relative values of those options across time. The theory uses a scaled version (Shahan & Craig, 2017) of the temporal weighting rule (e.g., Devenport & Devenport, 1994) to generate a series of weightings applied to experiences in past sessions such that

(1) $$w_{x}=\frac{1/t_{x}^{c}}{\sum_{i=1}^{n}1/t_{i}^{c}},$$

where *w*_*x*_ is the weighting for a particular session in the past and *t*_*x*_ is the time (i.e., number of sessions plus 1) between that session and the session of interest. The scaling exponent is calculated as *c =* λ*r* + 1, with *r* corresponding to the average running reinforcement rate for a particular response (i.e., target or alternative) across sessions, and λ is a free parameter (see Shahan & Craig, 2017, for details). The values of target and alternative behaviors are calculated by applying the appropriate series of weightings to reinforcement rates (i.e., *R*_*x*_) experienced across sessions for each option in a series of past sessions under consideration and summed across those sessions such that

(2) $$V=\sum_{x}w_{x}R_{x}.$$

Separate values thus obtained for the target (i.e., *V*_*T*_) and alternative (i.e., *V*_*Alt*_) behaviors are then used to calculate absolute response rates (in responses/minute) for the target (i.e., *B*_*T*_) and alternative (i.e., *B*_*Alt*_) behaviors:

(3) $$B_{T}=\frac{kV_{T}}{V_{T}+d_{1}\left(V_{\mathrm{Alt}}\right)+d_{0}\left(\frac{1}{A}\right)},$$

and

(4) $$B_{\mathrm{Alt}}=\frac{{\mathrm{kd}}_{1}\left(V_{\mathrm{Alt}}\right)}{\;V_{T}+d_{1}\left(V_{\mathrm{Alt}}\right)+d_{0}\left(\frac{1}{A}\right)},$$

where *k* is a free parameter scaling values into response rates and *A* represents the invigorating effects of the current values of the options and is calculated as *A = a*(*V*_*T*_ *+ V*_*Alt*_), with *a* as a free parameter scaling value to invigoration. The values calculated by Equation 2 and used in Equations 3 and 4 can be thought of as incorporating the longer term effects of the histories of reinforcement for target and alternative behaviors. Importantly for present purposes, the *d*_1_ and *d*_0_ terms serve to incorporate the influence of the more local signaling effects of alternative reinforcer deliveries or their absence, respectively. Specifically, *d*_1_ reflects the biasing effects (toward the alternative behavior) of the fact that alternative reinforcement availability signals the local absence of reinforcement for the target behavior. Similarly, *d*_0_ reflects the biasing effects (toward not responding) of the fact that the absence of alternative reinforcement signals that reinforcement is not available for either option. These biasing effects are assumed to develop with exposure to the relevant discriminative conditions using a simplified learning curve (Gallistel et al., 2004) such that

(5) $$d_{1}=d_{m}\left(1-e^{-x_{\mathrm{on}}}\right),$$

and

(6) $$d_{0}=d_{m}\left(1-e^{-x_{\mathrm{off}}}\right),$$

where *d*_*m*_ is a free parameter for a shared asymptotic value of *d*_1_ and *d*_0_, and *x*_*on*_ and *x*_*off*_ are increasing sessions of exposure to alternative reinforcement present (i.e., “on”) or absent (i.e., “off”). In a previous application of RaC2, the estimated value of *d*_*m*_ was higher for CDT (i.e., on/off alternative reinforcement) than for a condition arranging consistently available (i.e., all on) alternative reinforcement (Shahan, Browning, & Nall, 2020).

Again, assuming that “on” sessions, as signaled by the availability of alternative reinforcement, might be considered the relevant S+ periods and “off” sessions, as signaled by the absence of alternative reinforcer deliveries, might correspond to the relevant S− periods, then reductions in the duration of “off” sessions might be expected to reduce the asymptotic biasing effects resulting from this discrimination (i.e., *d*_*m*_). Further, based on the findings of Kalmbach et al. (2019) that discrimination of an auditory stimulus increases as a linear function the ratio of S−/S+ durations, *d*_*m*_ might reasonably be expected to decrease linearly with decreases in the duration of “off” sessions with a fixed “on” session duration.

**FIGURE 6:**
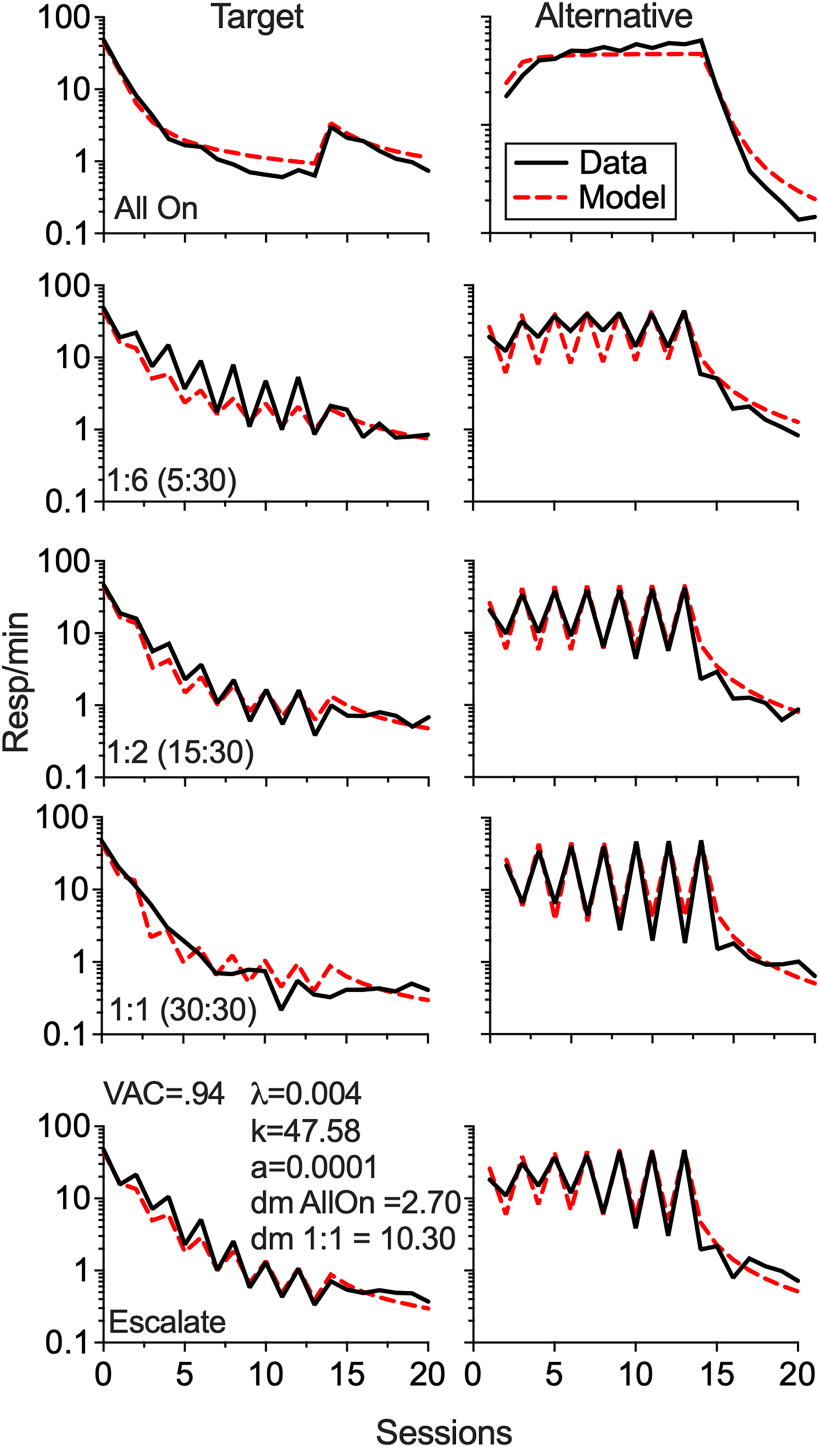
Fit of RaC2 to target and alternative behavior for all groups. Data are from the end of Phase 1 (presented on the *y*‐axis above zero) and across all sessions of Phases 2 and 3. Solid black lines represent means for each group, and dotted red lines represent model predictions. The left column shows target response rates. The right column shows alternative response rates. Note the logarithmic *y*‐axes.

Figure 6 shows the simultaneous fit of RaC2 to target and alternative behavior from the present experiment. Best fitting functions generated by the model were obtained via least‐squares regression (Microsoft Excel Solver) to log‐transformed data (see Shahan, Browning, & Nall, 2020, for discussion). The fit involved five free parameters, *k*, *a*, λ, *dmAllOn*, and *dm1:1* and involved 205 data points. Based on the assumption that *d*_*m*_ might be expected to decrease linearly with decreases in the off:on ratio, the values of *d*_*m*_ for the CDT 1:6, CDT 1:2, and CDT Esc groups were interpolated from the single line relating the values of *d*_*m*_ for off:on ratios of 0 (i.e., for the “All On” group) and 1 (i.e., for the CDT 1:1 group). During the fit, *dmAllOn* and *dm1:1* varied as free parameters and squared residual minimization was based on predictions generated by the full range of interpolated *d*_*m*_ values. Based on the final obtained estimates of *dmAllOn* (i.e., 2.70) and *dm1:1* (i.e., 10.30), the estimated line relating *d*_*m*_ to the off:on ratio was *d*_*m*_ = 7.60*x* + 2.70. For the CDT groups with a fixed “off” session duration, the same *d*_*m*_ value was used across all sessions (e.g., *d*_*m*_ for CDT 1:2 = 7.60 × 0.5 + 2.70 = 6.50). For the CDT Esc group, the value of *d*_*m*_ used for a particular session was similarly obtained based on the off:on ratio arranged in that session (see Appendix B for a fuller consideration of *d*_*m*_ estimates).

Overall, using this assumption of a linear relation between *d*_*m*_ and the off:on session durations, the fit accounts for 94% of the variance and captures most features of the data well. Despite the good description of the data, there is some obvious room for improvement. For example, the fit tends to underpredict target response rate increases and alternative response rate decreases observed during “off” sessions across Phase 2 for the CDT 1:6 group and to overpredict cycling of target response rates during Phase 2 for the CDT 1:1 group. Nevertheless, overall, the fit suggests that RaC2 can provide a reasonable description of how variations in “off” session durations affect treatment and subsequent resurgence, with the assumption that “off” session durations function comparably to how variations in S− durations affect other types of discriminations.

Given the success of this conceptualization, it could prove especially fruitful to identify other factors affecting discriminability of the relevant contingencies that might similarly be altered in ways that improve the clinical practicality of CDT procedures while maintaining the resurgence‐mitigating effects of the approach. The present study adds to a growing number of recently evaluated and innovative approaches to implementing CDT, including reducing the number of on/off cycles (Shahan et al., 2024) and arranging cycles of higher and lower magnitude alternative reinforcement (Ritchey et al., 2025). These and other CDT procedures that attempt to balance clinical practicality while preserving resurgence‐mitigation effectiveness will be uniquely suited for translation and evaluation in more applied contexts.

## AUTHOR CONTRIBUTIONS

**Shahan:** Conceptualization, formal analysis, funding acquisition, methodology, supervision, visualization, writing: original draft, review, and editing. **Hiltz:** Investigation, methodology, writing: original draft, review, and editing. **Avellaneda:** Formal analysis, investigation, methodology, software, writing: review and editing. **Greer:** Conceptualization, funding acquisition, methodology, writing: review and editing.

## CONFLICT OF INTEREST STATEMENT

The authors declare no conflicts of interest.

## ETHICS APPROVAL

All experimental procedures were conducted in accordance with the university's Institutional Animal Care and Use Committee.

## Figures and Tables

**FIGURE B1:**
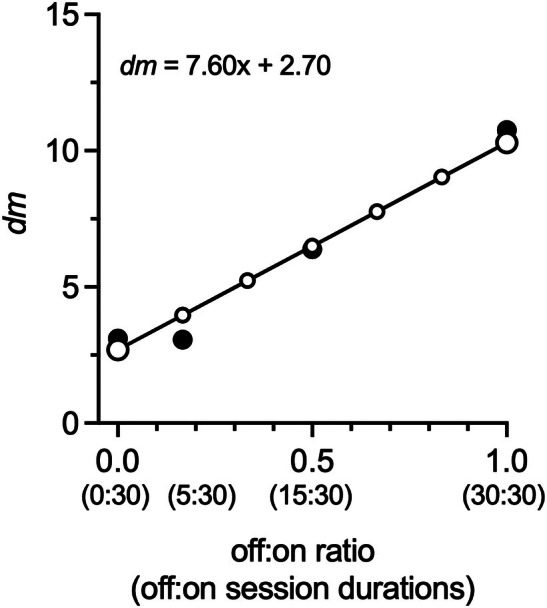
Linear relation between *d*_*m*_ values and off: on session duration. The line, equation, and empty symbols represent the linear relation used for *d*_*m*_ values in the fit of RaC2 presented in Figure 6. The filled symbols represent *d*_*m*_ values when that parameter was allowed to vary freely in a separate fit to all the groups exposed to a fixed off: on ratio.

## Data Availability

The data that support the findings of this study are available from the corresponding author upon reasonable request.

## Footnotes

^1^
A randomized clinical trial examining the efficacy of CDT with equal duration on/off sessions in reducing resurgence of severe problem behavior is currently underway (ClinicalTrials.gov, NCT05537610).

^2^
Male rats were used because a previous comparison of males and females in a resurgence experiment (Shahan, Browning, & Nist, 2020) found that although females responded at an overall lower rate, similar patterns of responding were obtained for the two sexes during treatment and resurgence tests. Using the same sample size as here but likely consisting of higher rate males and lower rate females could result in an overall increase in variability, a likely bimodal distribution, and a potential loss of power. Given that the overall patterns of responding were similar for the sexes and that the theoretical model fits generated similar parameter estimates (with the exception of asymptotic response rates), males were selected for use because their higher overall response rates could make it easier to detect smaller differences in resurgence in the conditions examined here.

^3^
Using the total time spent in extinction in Phase 2 produces the same relation, differing in terms of the rate constant for the exponential function.

## APPENDIX B

Figure B1 shows the linear relation used to determine *d*_*m*_ values for the fit of RaC2 reported in Figure 6 of the main text. The equation for the line was derived by allowing the *d*_*m*_ values for the All On (i.e., *dmAllOn*) and CDT 1:1 (*dm1:1*) groups to vary as free parameters during the fit. These fitted parameters are represented in Figure B1 by the larger open circles anchoring the left and right endpoints of the line. The *d*_*m*_ values for the other off:on ratios used for all sessions for the remaining CDT groups and for the escalating off:on ratios across “off” sessions for the escalate group are represented by the small open circles. Assuming such a linear relation reduced the number of *d*_*m*_ parameters for the fit from 10 to 2, as the slope and intercept of the line are fully determined by the fitted values of *dmAllOn* and *dm1:1*. The quality of the fit in Figure 6 based on the assumed linear relation is consistent with the suggestion that discrimination of the reinforcement contingencies trained during CDT varies linearly with the off:on session duration ratio in a manner similar to the effect of the S−/S+ ratio on discrimination of arbitrary stimuli (e.g., Kalmbach et al., 2019).

Because *d*_*m*_ values serve as a proxy for discriminability of the reinforcement contingencies arranged by CDT, it might also be of interest to examine how *d*_*m*_ values vary with the off:on ratio when *d*_*m*_ is allowed to adjust as a free parameter across groups in a fit of RaC2 rather than assuming that it increases linearly with the off:on ratio. The filled symbols in Figure B1 represent the obtained values of *d*_*m*_ from a fit of RaC2 to the four groups with a fixed off:on session duration (other obtained parameters of the fit *k*, *a*, and λ varied marginally but not meaningfully from those reported in Figure 6). The escalate group was not included in this fit because its inclusion would have necessitated estimation of six additional free parameters and each off:on ratio was only in effect for this group for a single session (as opposed to the entirety of Phase 2 for the other groups)—a fact likely to lead to poor estimation of the asymptotic discriminability *d*_*m*_ represents. The figure shows that estimated *d*_*m*_ values do indeed increase with the off: on ratio, and they do so roughly linearly.
